# Targeting Lactylation for Cancer: Mechanisms, Effects, and Therapeutic Prospects

**DOI:** 10.3390/ijms262311278

**Published:** 2025-11-21

**Authors:** Dong Chang, Daolong Li, Yuxi Sun, Jiekang Shi, Shengping Zhang, Chuangui Wang

**Affiliations:** Biomedical Research Institute, School of Life Sciences and Medicine, Shandong University of Technology, Zibo 255000, China; 15695499940@139.com (D.C.); longdl2022@163.com (D.L.); 17560351231@163.com (Y.S.); 13386461146@163.com (J.S.)

**Keywords:** lactate, lactylation, metabolic, immune microenvironment, tumor

## Abstract

Recent studies reveal that lysine lactylation plays a pivotal and multifaceted role in tumor progression. Here, we provide a systematic overview of the mechanisms underlying lactylation, highlighting its regulation of tumor metabolic reprogramming and immune microenvironment remodeling. We further summarize how lactylation drives malignancy across diverse cancer types and discuss emerging strategies to therapeutically target lactylation in cancer. By integrating current findings, this review offers a comprehensive framework for understanding lactylation in tumor biology and identifies key gaps for future investigation, thereby providing a valuable reference for both basic research and therapeutic development.

## 1. Introduction

Lactylation is a post-translational modification initially discovered in 2019 [1]. This protein modification is widely present and highly conserved across various organisms [2,3,4,5,6,7,8,9,10,11,12,13,14,15,16,17]. The process of lactylation begins with the conversion of lactate into lactyl donors such as lactyl-coenzyme A (CoA) [1], lactyl-adenosine monophosphate (AMP) [18], and lactyl-glutathione (GSH) [19]. These lactyl donors then transfer the lactyl group to lysine residues on histones or non-histone proteins through lactyltransferases or non-enzymatic reactions, resulting in the lactylation of substrate proteins. Delactylases can reversibly remove the lactyl group. Lactylation plays a pivotal role in regulating gene transcription [20,21,22], protein function [23,24,25], and degradation [26,27]. Cancer is the second leading cause of disease worldwide. In 2023 alone, 18.5 million people were diagnosed with cancer, and 4.33 million died from the disease. Projections indicate that both cancer incidence and mortality will continue to rise sharply by 2050 [28]. Over the past six years, research has identified lactylation as a critical regulator of tumor metabolic reprogramming, microenvironment remodeling, and tumor malignant progression [18,29,30]. Thus, it is imperative to produce a comprehensive review of lactylation, providing a solid foundation for future research directions and offering a theoretical basis for utilizing lactylation as a potential therapeutic target in tumor treatment.

This review starts with a detailed overview of the three currently identified mechanisms of lactylation and their key components. We then clarify the functions of lactylation in regulating gene transcription and protein function. Additionally, we provide an in-depth account of specific histone lactylation sites, their effects, and the regulatory targets involved in various diseases. We also explore how lactylation impacts tumor biological processes such as metabolic reprogramming and microenvironment remodeling, with a particular emphasis on the tumor immune microenvironment and what molecular mechanisms lactylation promotes tumor malignant progression. Furthermore, we identify potential therapeutic targets for diseases by modulating lactylation. In conclusion, we discuss future prospects and challenges in lactylation research, offering cautious insights based on current studies and our own experience to provide valuable help for researchers in this field.

## 2. Exploring Lactylation: Modification Mechanism, Key Writers, Donors and Erasers

### 2.1. Three Types of Lactylation Mechanisms

**Figure 1:**
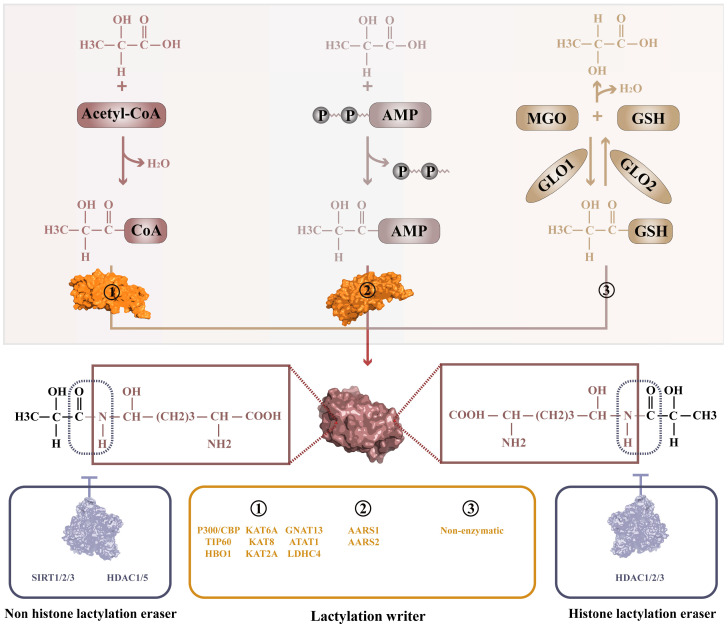
Three mechanisms of lactylation modification. The schematic illustrates three modes of lactylation. The yellow boxes denote the “writers” involved in each mode, while the two blue boxes indicate the “erasers” for histone and non-histone lactylation, respectively. Carriers of the lactyl group and other reaction components are shown as indicated. Arrows represent promotion of the corresponding steps, and “T” denotes inhibition. MGO: methylglyoxal; GLO1/2: glyoxalase 1/2; SIRTs: sirtuins; P300: EP300 Lysine Acetyltransferase; TIP60: lysine Acetyltransferase 5; HBO1: lysine acetyl transferase 7; KATs: lysine acetyltransferases; GNAT13: GNAT family of acetyltransferases 13; ATAT1: alpha tubulin acetyltransferase 1; LDHC4: lactate dehydrogenase C4; HDACs: histone deacetylases.

Three types of lactylation modifications have been identified: (1) lysine acyltransferases (KATs)-mediated lactylation, where KATs function as lactyltransferases (writers) with lactyl-CoA serving as the acyl donor; (2) alanyl-tRNA synthetase (AARS)-mediated lactylation, where AARS act as lactyltransferases with lactyl-AMP as the acyl donor; and (3) non-enzymatic lactylation, where lactyl-GSH acts as the lactyl donor (Figure 1).

#### 2.1.1. Lactylation with KATs as the Main Writers and Lactyl-CoA as the Donor

In the field of lactylation, P300 (KAT3B) is the most extensively studied lactyltransferase. Substantial evidence has shown that P300 plays a crucial role in the lactylation of specific histone sites, including histone H3 lysine 18 (H3K18) [1,31,32,33], H3K9 [34], and H4K12 [35], using lactyl-CoA as the lactyl donor (Figure 1). P300 also lactylates non-histone proteins such as the snail family transcriptional repressor 1 (SNAIL1) [36], YY1 transcription factor (YY1) [37,38] and so on. The highly homologous CBP often collaborates with P300 in lactylation under specific conditions [36,39]. Additionally, CBP (KAT3A) has been shown to function as an independent lactyltransferase, as demonstrated by its role in mediating the lactylation of MRE11 homolog, double strand break repair nuclease (MRE11) during DNA damage [30].

Members of the MYST (MYST family of histone acetyltransferases) family have also been implicated in lactylation functions. TIP60 (KAT5) has been observed to mediate the lactylation of nibrin (NBS1) [29] and phosphatidylinositol 3-kinase catalytic subunit type 3 (VPS34) [40], while another family member, HBO1 (KAT7), has been characterized as lactylating H3K9 [41]. Both enzymes use lactyl-CoA as the donor to transfer the lactyl group to their respective substrates. Furthermore, molecular docking predicts that lysine acetyltransferase 6A (MOZ, also known as KAT6A) and lysine acetyltransferase 8 (MOF, also known as KAT8) have a high affinity for lactyl-CoA [41]. Currently, there are no reports of lactylation associated with lysine acetyltransferase 6B (MORF, also known as KAT6B).

Recent studies have also suggested that certain members of the GNAT family mediate lactylation modifications. For example, interleukin 1 beta (IL1β)-dependent GCN5 (also known as KAT2A) lactylates H3K18 in mice [40]; GNAT13 acts as a lactyltransferase in the cariogenic bacterium Streptococcus mutans and has been shown to lactylate RPOA in vitro [12]; ATAT1 lactylates N-acetyltransferase 10 (NAT10) in Kaposi’s sarcoma-associated herpesvirus (KSHV), facilitating KSHV reactivation [42]. The latest research shows LDHC4 promotes the lactylation of acetyl-CoA acyltransferase 2 (ACAA2) at K214, enhancing its activity and increasing free fatty acid accumulation [43]. However, these reports do not elucidate the specific mechanisms by which these lactyltransferases function, suggesting the need for further investigation.

#### 2.1.2. Lactylation Mediated by AARS, with Lactyl-AMP as the Donor

It has been suggested that AARS also acts as a lactyltransferase [18,23,44]. AARS is an evolutionarily conserved protein that primarily catalyzes the formation of alanyl-tRNA during cytoplasmic or mitochondrial protein synthesis. Recently, AARS has also been revealed to mediate alanylation modifications on proteins [45], and it is noteworthy that alanine and lactate exhibit substantial structural resemblance. A recent study reported that AARS1 utilizes lactate and ATP as substrates to initially synthesize lactyl-AMP, which then serves as a donor to transfer the lactyl group onto substrates such as tumor protein P53 (P53) [18]. Another study found that AARS1 lactylates the YAP/TEAD1 complex in gastric cancer (GC) cells using the same catalytic mechanism [44]. In mitochondria, hypoxia induces the accumulation of mitochondrial AARS2, which also uses ATP and lactate as substrates to lactylate pyruvate dehydrogenase E1 subunit alpha 1 (PDHA1) and carnitine palmitoyl transferase 2 (CPT2), thereby limiting oxidative phosphorylation (OXPHOS). The precise mechanism by which AARS2 exerts its effects remains elusive [23]. However, given the high degree of conservation observed in AARS, it is plausible that AARS2 employs a catalytic mechanism akin to that of AARS1 to perform its lactylation function.

#### 2.1.3. Non-Enzymatic Lactylation

There is a distinctive non-enzymatic lactylation process that utilizes methylglyoxal as a substrate [19]. Specifically, the glycolysis byproduct methylglyoxal rapidly combines with GSH under the catalysis of GLO1 to form lactyl glutathione (LGSH). The lactyl group on LGSH can directly modify proteins such as some glycolytic enzymes through D-lactylation. LGSH can also be hydrolyzed by GLO2 into GSH and D-lactate, which can then be further utilized by the cell [19] (Figure 1). It is important to note that the concentration of methylglyoxal is very low under normal physiological conditions; however, it increases significantly under hyperglycemic conditions [46]. Furthermore, the non-enzymatic reaction described above leads to D-lactylation of substrate proteins. In contrast to the majority of lactylation modifications discussed thus far, which involve L-lactylation using L-lactate as the substrate, recent reports indicate that D-lactylation and non-enzymatic lactylation modifications can occur when the glycolytic pathway is inhibited or when the glyoxalase system is dysfunctional [47]. Accordingly, research into this type of non-enzymatic lactylation must take into account the specific research conditions under which it is conducted.

### 2.2. Lactylation Erasers

A total of 18 deacylases are encoded by the human genome, which can be classified into two major classes: Zn^2+^ dependent HDACs and NAD+ dependent SIRTs. As classical deacylases, HDACs can be further categorized into Class I (HDAC1/2/3/8), Class IIa (HDAC4/5/7/9), Class IIb (HDAC6/10), and Class IV (HDAC11). These HDACs have distinct subcellular localization characteristics, which largely determine their substrate specificity [48]. SIRTs, also known as Class III HDACs, are distributed across various cellular compartments, including the cytoplasm (SIRT2), mitochondria (SIRT3/4/5), nucleus (SIRT1/6), and nucleolus (SIRT7) [49].

#### 2.2.1. Histone Delactylation Eraser

**Figure 2:**
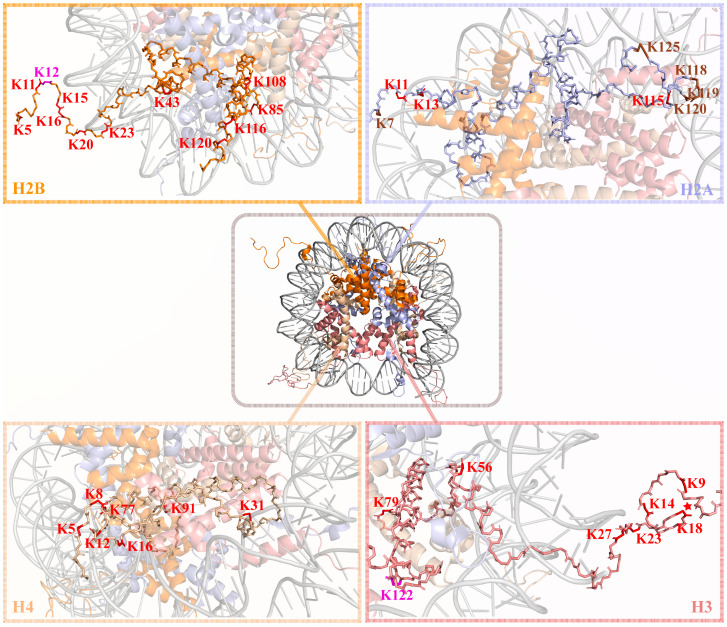
Lactylation sites of histones. The magnified views of histones H2A, H2B, H3, and H4 are shown in purple, orange, pink, and light yellow boxes, respectively, with all currently reported lactylation sites detected by mass spectrometry highlighted. Sites initially identified in earlier studies are marked in different colors: red for the earliest discoveries, magenta for later reports, and brown for more recent findings. The three-dimensional structure of the histone octamer and DNA was obtained from the PDB (https://www.rcsb.org/structure/3LEL (accessed on 25 August 2024)) and modified using PyMOL 2.5.5. To enhance clarity, the magnified sections have been rotated at specific angles. Lactylation has been experimentally confirmed at key histone sites, such as H3K9, H3K18, and H4K5. Among these, H3K18 remains the most extensively studied site of lactylation.

It has been found that many sites on tumor histones can be lactylated (Figure 2 and Table 1). Class I HDACs primarily catalyze the de-L-lactylation of histones. Evidence suggests that the major members of Class I HDACs, particularly HDAC1/2/3, exhibit strong de-L-lactylation activity in vitro. This finding was later confirmed by experiments showing that HDAC1/2/3 can de-lactylate H3K18 and H4K5 [50].

#### 2.2.2. Non-Histone Delactylation Eraser

The delactylation of non-histone proteins is mainly mediated by SIRT1, SIRT2, SIRT3, and HDAC3. SIRT1 is involved in the delactylation of high mobility group box 1 (HMGB1) in macrophages [39] and regulates pyroptosis via the delactylation of canopy FGF signaling regulator 3 (CNPY3) [59]. Additionally, SIRT1 participates in the delactylation of proteins in the hippocampus of aged mice [60]. SIRT2, a key delactylase, engages in non-enzymatic lactylation reactions [61] and promotes the delactylation of methyltransferase 16, RNA N6-adenosine (METTL16) at K229 [62]. SIRT3 mediates the delactylation of proteins such as PDHA1 [63], CPT2 [23], and Cyclin E2 (CCNE2) [64]. HDAC3 has been identified as a mediator of DNA damage repair through the delactylation of NBS1 [29], suggesting that HDACs may also have the ability to delactylate non-histone proteins. Elevated endogenous lactate in radioresistant TNBC cells promoted DNA repair via MRE11 Lys673 lactylation, a critical modification conferring radioresistance, HDAC5 was identified as the key delactylase for MRE11 Lys673, validated by HADDOCK docking [30]. Other SIRTs have been shown to mediate various types of post-translational modifications. Some representative non-histone lactylation information in tumors is shown in Table 2.

## 3. Lactylation Regulates Tumor Metabolic Reprogramming and Microenvironment Remodeling

### 3.1. Crosstalk Between Lactylation and Tumor Metabolic Reprogramming

**Figure 3:**
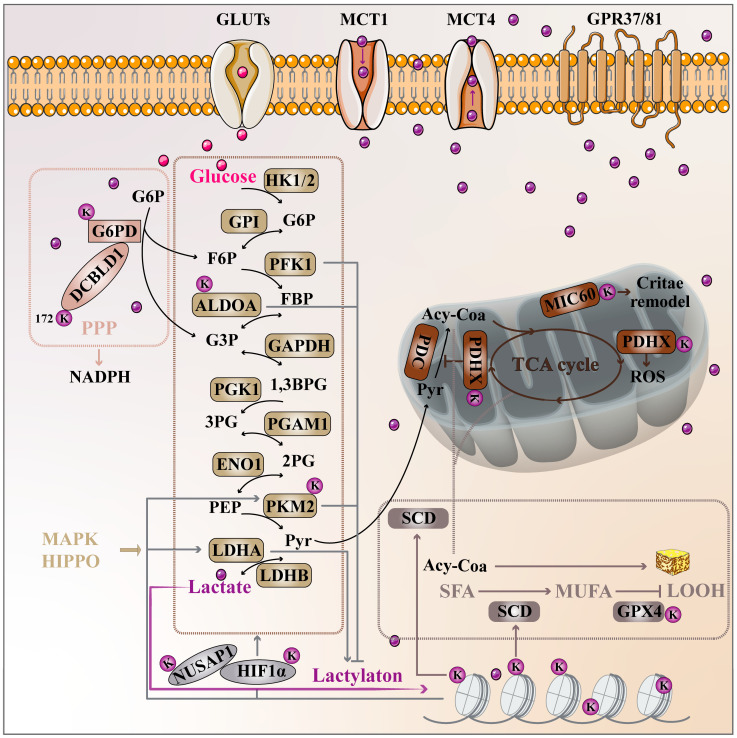
Crosstalk between lactylation and metabolism in tumors. Lactylation is involved across the full spectrum of tumor metabolism including glycolysis, gluconeogenesis, lipid metabolism, and mitochondrial metabolism (each delineated by dashed boxes). On one hand, histone lactylation reprograms tumor metabolism by promoting enzymes within these pathways, such as LDHA, PKM2, and SCD; on the other hand, lactate generated by glycolysis or imported into tumors via channels such as MCT1 and GRP78 can directly modify proteins along these metabolic routes. Oncogenic signaling pathways, including MAPK and HIPPO, also exert profound effects on lactylation events linked to tumor glucose metabolism. Purple spheres denote lactate; purple spheres labeled “K” mark lactylated lysine residues on proteins; pink spheres denote glucose. Arrows indicate promotion of the corresponding reactions, and “T” denotes inhibition.

A substantial body of research highlights lactylation’s critical role in regulating metabolic reprogramming across tumors. On one hand, dysregulated glycolysis in a tumor leads to increased lactate production. Excessive lactate can enhance the transcription of enzymes involved in glycolysis and other metabolic processes by inducing lactylation at specific histone sites. On the other hand, when intracellular lactate concentration elevated intracellular lactate concentration-whether from endogenous production or external uptake-lactate acts as a lactyl group donor. Lactyltransferases then directly modify key enzymes involved in glycolysis, gluconeogenesis, and other metabolic pathways, thereby driving tumor metabolic reprogramming (Figure 3). In the following sections, we will provide a detailed explanation of how lactylation mediates the reprogramming of tumor metabolic processes such as glycolysis through these two mechanisms. Given that lactate synthesis and transport mechanisms have been thoroughly reviewed [70], this review focuses on studies that specifically report and elucidate the crosstalk between lactylation and tumor metabolic reprogramming.

#### 3.1.1. Lactylation and Glycolysis

Lactylation emerged as a central player in the metabolic reprogramming of cancer cells, particularly in glycolysis. Histone lactylation directly connects glycolytic reprogramming to epigenetic regulation, enabling the maintenance of a cancerous phenotype. In hepatocellular carcinoma (HCC), H2B-K58 lactylation at the NDRG1 locus links the activity of LDHA to epigenetic control, creating a feedback loop that supports senescence-resistant clones [71]. The modification of histones, like H3K18, also regulates glycolysis in lung cancer, where it activates YTHDF2, enhancing glycolysis and cancer stemness through m6A-modified mRNA encoding SFRP2 [72].

On the non-histone side, lactylation modifies key metabolic enzymes and proteins that drive glycolysis. LDHA, a central enzyme in glycolysis, is a primary target for lactylation in multiple cancers. In colorectal cancer (CRC), LDHA is activated by KRAS^G12D^-induced MEK/ERK signaling, increasing lactate production. This lactate lactylates GCLM, enhancing ferroptosis resistance [73]. Furthermore, lactate driven PKM2 lactylation amplifies glycolysis in pancreatic cancer, supporting tumor cell survival and promoting metastasis by facilitating epithelial–mesenchymal transition [74]. Interestingly, ADLOA lactylation in tumor cells suppresses its enzymatic activity, which in turn inhibits glycolytic flux [75]. In HCC, lactate-induced lactylation of IGF2BP3 enhances the m6A modification of PCK2 and NRF2 mRNAs, creating a circuit that drives lenvatinib resistance [76]. This lactylation driven metabolic reprogramming highlights how non-histone lactylation supports the reprogramming of glycolytic pathways, making them more efficient and resilient. It is worth mentioning that 6PD lactylation in lung cancer enhances the pentose phosphate pathway (PPP), further supporting tumor cell proliferation and migration [77].

In addition to enzymes, lactylation also influences glycolytic metabolism by targeting key metabolic regulators. For instance, NUSAP1, a protein involved in glycolysis, interacts with c-MYC/HIF-1α at the *LDHA* promoter. Here, lactate-driven lysine lactylation prevents NUSAP1 degradation, locking a feed-forward loop that sustains glycolysis and drives metastasis in pancreatic cancer [26]. In CRC, lactagenesis, driven by ALDOB-PDK1, increases extracellular lactate, activating CEACAM6 and sustaining chemoresistance [78].

Lactate itself is a key mediator of these lactylation events, acting as both a metabolic substrate and a signaling molecule that modulates the activity of glycolytic enzymes. In GC, lactate-induced VPS34 lactylation strengthens autophagic flux, enabling cells to survive under nutrient deprivation [40]. In CRC, lactate facilitates GPR37-mediated activation of the Hippo pathway, upregulating LDHA, further promoting glycolysis and histone lactylation at H3K18, which fosters a pro-metastatic niche [79].

Together, these findings underscore the intimate relationship between glycolytic metabolism and lactylation, revealing how both histone and non-histone lactylation serve as central regulators of the tumor metabolic reprogramming process. The orchestration of glycolysis via lactylation not only enhances metabolic flexibility in response to nutrient stress but also drives the Warburg effect, metastasis, immune evasion, and therapeutic resistance in cancer.

#### 3.1.2. Lactylation and Lipid Metabolism

Lactylation has become a critical post translational modification that reshapes lipid metabolism in cancer. By modifying key enzymes and regulators, lactylation drives lipid metabolic reprogramming that supports tumor progression and survival under stress. A prime example is lactylation’s impact on YTHDC1, which stabilizing NEAT1 via m6A modification of its mRNA, leading to the recruitment of p300 and activation of SCD, a key enzyme in lipid desaturation. This rewires lipid biosynthesis, fueling cancer progression [80]. Additionally, GPX4 lactylation, induced by AGEs–EGFR/SRC signaling. It stabilizes GPX4, protecting cells from oxidative stress and preventing ferroptosis. Breviscapine, a flavonoid that targets this pathway, destabilizes GPX4, promoting lipid peroxidation [81]. Lactylation also regulates ACSL4 and ACLY in lipid synthesis. In pancreatic cancer, PSMD14 mediated de-ubiquitination of LDHA leads to elevated lactate levels, which activate ACLY via H3K18 lactylation. This enhances lipid synthesis, promoting cancer progression [82]. In lung cancer, SUMO2 lactylation of ACSL4 impairs its function, promoting ferroptosis resistance. By enhancing ACSL4 degradation, lactylation helps maintain lipid homeostasis and protects cells from oxidative damage [83]. Finally, HDAC2-mediated removal of PD-L1 K189 lactylation triggers vimentin-assisted nuclear import, enhances YY1-dependent SQLE transcription and cholesterol biosynthesis, and accelerates tumor growth [84]. Overall, lactylation regulates key enzymes in lipid synthesis, desaturation, and turnover, enabling tumors to adapt to metabolic stress, resist cell death, and sustain proliferative capabilities.

#### 3.1.3. Lactylation and Mitochondrial Metabolism

Lactylation plays a pivotal role in regulating mitochondrial metabolism. One critical mechanism involves p300-mediated acetylation of PDHX at K488, which disrupts the pyruvate dehydrogenase complex (PDC), rerouting carbon flux toward aerobic glycolysis. This shift increases lactate production and sustains oncogenic transcription, particularly through H3K56 lactylation [85]. SIRT3-mediated delactylation of ME2 at K352 regulates mitochondrial redox balance by dampening ME2 activity. This disruption of NADPH levels reduces tumor growth, illustrating how reversible lactylation serves as a potential drug target for controlling metabolic pathways that fuel cancer cells [86]. Additionally, DAPK2 dysfunction leads to MIC60 lactylation, triggering mitochondrial metabolic reprogramming that promotes resistance to EGFR-TKI therapies and facilitates metastasis. This lactylation modification influences mitochondrial function, further demonstrating the critical role of lactylation in driving metabolic changes [87]. Together, these studies underscore how lactylation intersects with mitochondrial metabolism, influencing critical processes such as glycolysis, redox balance, and cristae remodel.

### 3.2. Lactylation in Tumor Microenvironment Remodeling

**Figure 4:**
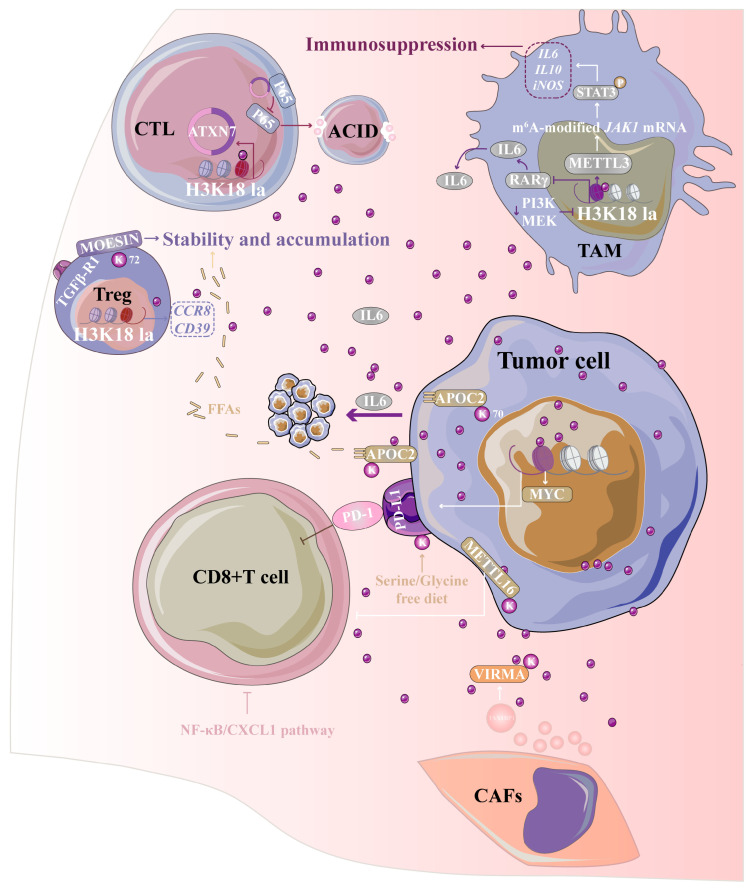
Lactylation in tumor microenvironment (TME) remodeling, particularly the immune compartment. Lactate secreted by tumor cells and other TME-resident cells is a principal driver of this remodeling. In tumor cells, lactylated PD-L1 strengthens its interaction with T cells, fostering an immunosuppressive milieu; additionally, APOC2-dependent release of FFAs further contributes to immune suppression. Lactylation of proteins in CAFs and TAMs—such as VIRMA and histone H3K18—also promotes TME remodeling. Purple spheres denote lactate; purple spheres labeled “K” mark lactylated lysine residues on proteins; short yellow cylinders represent FFAs. Arrows indicate promotion of the indicated processes, and “T” denotes inhibition.

Lactylation plays a critical role in the tumor microenvironment (TME) by regulating immune evasion and tumor progression through the modulation of key pathways in various cell types. It primarily impacts immune responses by altering the function of T-cells and macrophages, as well as influencing the interactions between tumor cells and stromal cells, such as cancer-associated fibroblasts (CAFs) (Figure 4).

#### 3.2.1. Lactylation and Macrophage

Lactylation was initially identified as a key factor in immune microenvironment remodeling through its role in macrophage polarization. Increased histone lactylation in the late phase of M1 macrophage polarization induces the transcription of homeostatic genes involved in wound healing, such as Arg1 [1]. Lactylation modulating macrophage polarization toward the M2 phenotype, which contributes to immune suppression and tumor progression across various cancers. In HCC, the splicing factor SRSF10 stabilizes MYB mRNA, amplifying a glycolysis-lactate-H3K18la feed forward loop. This loop polarizes macrophages to the M2 state, undermining the effectiveness of PD-1 blockade [88]. In CRC, tumor-intrinsic PCSK9 enhances PI3K/AKT signaling and promotes epithelial–mesenchymal transition (EMT), which drives lactate accumulation and lactylation. This process elevates MIF, skewing macrophages toward an M2 phenotype and reinforcing a pro-metastatic, immune-suppressive niche that supports tumor progression and metastasis [89]. In pancreatic cancer, lactylation initiates several immune-modulatory feedback loops. Histone lactylation at H3K18 upregulates ACAT2, which acetylates and stabilizes MTCH2, thereby suppressing OXPHOS and amplifying lactate production. This metabolic shift, in turn, polarizes tumor-associated macrophages (TAMs) toward the M2 phenotype via small extracellular vesicle (sEV)-delivered cholesterol [90]. Lactate also induces ENSA K63 lactylation, triggering STAT3/CCL2 signaling to recruit and reprogram TAMs, further contributing to therapeutic resistance [91].

Moreover, VSIG4^+^ TAMs rely on a lactate-H3K18la-STAT3-SPP1 axis to sustain neutrophil crosstalk and impair antigen presentation, thus maintaining a suppressive immune environment [92]. Lactate generated by CRC cells promotes H3K18 lactylation in tumor-infiltrating myeloid cells (TIMs) and TAMs, inhibiting RARγ transcription in TAMs or enhancing methyltransferase 3, N6-adenosine-methyltransferase complex catalytic subunit 3 (METTL3) transcription in TIMs. This activation of the IL6-JAK-STAT axis endows TAMs and TIMs with functions that promote CRC progression [93,94]. Finally, in prostate cancer (PC), dual inhibition of PI3K and MEK pathways reduces lactate production, suppresses H3K18 lactylation in TAMs, and restores their phagocytic activity [95].

#### 3.2.2. Lactylation and T Cell

Lactylation by modulating T-cell function across various cancer types. Histone lactylation, in particular, has been shown to regulate immune checkpoints such as PD-L1, contributing to the suppression of CD8^+^ T-cell activity and the promotion of tumor progression. In HCC, lactylation enhances the expression of major vault protein (MVP), which inhibits the β-TrCP-mediated degradation of PD-L1, stabilizing PD-L1 and effectively suppressing CD8^+^ T-cell function [96]. Similarly, in GC, lactylation facilitates immune escape by stabilizing PD-L1 through the activation of the PI3K/AKT/HIF1α pathway, which enhances glycolysis and lactate production [97]. In CRC, a serine/glycine-free diet has been shown to enhance CD8^+^ T-cell accumulation but simultaneously promote PD-L1 lactylation, which enables immune evasion. Importantly, this immune suppression can be reversed by PD-1/PD-L1 blockade, highlighting the intersection of metabolic factors and immune regulation [98]. Beyond lymphocytes, lactylation also influences the innate immune compartment. In the CRC microenvironment, the loss of Claudin-7 in epithelial barriers triggers NF-κB/CXCL1 activation, which reprograms neutrophils and impairs CD8^+^ T-cell function, further reducing the effectiveness of immunotherapy [99]. Similarly, in lung cancers, lactylation modulates immune escape by activating key signaling pathways. In non-small cell lung cancer (NSCLC), lactylation at H3K18 activates the POM121/MYC/PD-L1 axis, leading to diminished CD8^+^ T-cell activity and enhanced immune evasion [100]. Similarity, lactate-induced H3K18 lactylation activates Nur77, further impairing T-cell function and promoting immune evasion [101]. Additionally, non-histone lactylation plays a critical role in lung cancer progression. In NSCLC, lactate induces lactylation of APOC2 at K70, which enhances its stability. This process leads to the release of free fatty acids (FFAs), an accumulation of regulatory T cells (Treg) in the TME, and resistance to anti-PD1 immunotherapy. Notably, using a custom anti-APOC2 K70-lac antibody can sensitize tumors to anti-PD1 immunotherapy [102]. Lactate secreted by the tumor into the surrounding environment enhances the lactylation of moesin at K72, thereby amplifying transforming growth factor beta (TGFβ)- SMAD family member (SMAD) signaling and further stabilizing Treg cells, ultimately leading to the formation of an immunosuppressive microenvironment around HCC [103]. Finally, lactate sensing in tumor-associated Schwann cells in pancreatic ductal adenocarcinoma (PDAC) induces METTL16 K269 lactylation, stabilizing m6A-dependent CTCF and activating immunosuppressive ligands. This metabolic stress further weakens CD8^+^ T-cell function and contributes to PD-1 resistance [104].

#### 3.2.3. Lactylation and Other Immune Cells

Lactylation also engages in complex interactions with other immune cells. In the context of B cell programs, both EP300 and HDAC1–3 are upregulated in HCC, with HDAC1/2 mediated immune infiltration—particularly involving B-cell responses—correlating with poor prognosis [105]. In CRC, particularly in microsatellite instability-high (MSI-H) cases, the recruitment of EP300 by MNDA to the CXCR2 promoter leads to H3K18 lactylation, driving the infiltration of polymorphonuclear myeloid-derived suppressor cells (PMN-MDSCs). This process confers primary resistance to PD-1 therapy, highlighting lactylation as a critical mechanism of immune suppression that can potentially be reversed therapeutically [106]. Additionally, in TIMs, lactate sensing via H3K18 lactylation upregulates METTL3, which installs m6A modifications on JAK1 mRNA, enhances JAK1–STAT3 signaling, reinforcing the immunosuppressive activity of myeloid cells and establishing lactylation-driven RNA modification as a dominant axis for immune evasion [93].

#### 3.2.4. Lactylation and CAFs

Lactylation plays a crucial role in regulating cancer progression by modulating the interaction between CAFs and tumor cells. A key mechanism involves the secretion of lactate by CAFs, which induces various molecular modifications in both CAFs and tumor cells, influencing the dynamics of the TME. In CRC, CAF-derived lactate induces ANTXR1 lactylation at K453, stabilizing the protein and activating RhoC/ROCK1/SMAD5 signaling, which promotes cancer stemness and resistance to oxaliplatin [107]. Furthermore, lactylation accelerates CAF proliferation, migration, and invasion while upregulating COL1A1, a marker that correlates with poor prognosis [108]. In lung cancer, CAF-secreted lactate induces EMT through H3K18 lactylation at the METTL3 promoter, enhancing tumor progression and contributing to resistance against EGFR-TKIs through the CTHRC1-glycolysis-H3K18la feedback loop [109]. In GC, CAF-secreted LOX triggers TGFβ-IGF1 signaling, which accelerates EMT and glycolysis. Concurrently, lactate-induced H3K18 lactylation directly drives PD-L1 transcription, undermining the effectiveness of PD-1/PD-L1 therapy [110]. Moreover, CAFs also release sEV containing circTAX1BP1, which induces VIRMA lactylation at K1713. This modification stabilizes SP1 mRNA, driving TGFβ transcription and further promoting EMT [111]. These findings collectively highlight the central role of lactylation in modulating the CAF–tumor interaction.

## 4. Lactylation Promotes Malignant Progression of Tumors

The role of lactate in the TME and metabolic reprogramming has been extensively and authoritatively summarized [70,112,113] and will not be elaborated here. We will primarily focus on summarizing the role of lactylation in proteins driving the malignant progression of tumors and chemotherapy resistance, thereby revealing the specific role of lactate as a substrate for lactylation in tumorigenesis and progression.

Genome instability is one of the key characteristics of cancer [114] and most chemotherapeutic agents targeting tumors induce DNA damage, surviving cells must rapidly repair this DNA damage to ensure their survival [115]. The MRE11-RAD50-NBS complex is a major participant in this process [116], and studies have shown that lactylation plays a crucial role in it. Lactate-induced lactylation of NBS1 K388 and MRE11 K673 enhances homologous recombination repair in tumor cells following DNA damage. Lactylation of NBS1 K388 is critical for the formation of the MRN complex and the accumulation of homologous recombination repair proteins at sites of DNA double-strand breaks, while lactylation of MRE11 K673 promotes its binding to broken DNA and the resection of damaged ends. TIP60 and HDAC3 mediate the lactylation and delactylation of NBS1, respectively, while CBP/P300 acts as the writer for MRE11 K673 [29,30]. The data indicate that using the LDHA inhibitor stridently or knocking out LDHA effectively inhibited NBS1 K388 lactylation, thereby improving chemotherapy resistance in tumor cells [29]. Additionally, small molecule peptides that specifically inhibit MRE11 K673 lactylation have been identified, and the use of these peptides significantly enhanced the cytotoxic effects of cisplatin and poly (ADP-ribose) polymerase 1 (PARP) inhibitors on tumors [30].

A pioneering discovery revealed that AARS1 functions atypically as a lactate sensor in tumor cells, catalyzing the lactylation of various proteins, including P53. Lactylation of P53 at K120 and K139 hinders its liquid–liquid phase separation and significantly weakens its ability to bind to DNA, thereby promoting tumorigenesis. Exogenously administered β-alanine can competitively bind to lactate with AARS1, inhibiting P53 lactylation and thus enhancing the efficacy of chemotherapy in tumors [18].

The following summarizes numerous examples of lactylation promoting malignant progression in various types of tumors.

### 4.1. Digestive System Cancers

#### 4.1.1. Colorectal Cancer

Lactylation plays a central role in driving CRC progression, with significant contributions to tumor proliferation, metastasis, invasion, and resistance to chemotherapy. This process involves the rewiring of cellular metabolism, DNA repair, and redox balance, each of which is critical for maintaining an aggressive and treatment-resistant tumor phenotype.

Chemoresistance is a major feature of lactylation’s role in CRC. The glycolysis inhibitor tsRNA-08614, targeting ALDH1A3, reduces H3K18 lactylation and EFHD2 transcription, effectively restoring oxaliplatin sensitivity [117]. In contrast, GOLPH3 enhances glycolysis and lactate-H3K18la positive feedback, fostering tumor growth and radioresistance [118]. The attenuation of SMC4 promotes glycolysis, raising lactate and histone lactylation, which increases ABC transporter expression and results in chemotherapy-insensitive, low-proliferative CRC clones [119]. Similarly, lactylation of ENO1 enhances glycolysis and promotes CRC malignancy through the NSUN2/YBX1/m(5)C-ENO1 positive feedback loop [67]. Additionally, tumor-derived lactate supports autophagy and hypoxic cell survival by upregulating RUBCNL, a protein involved in autophagosome maturation, contributing to resistance to bevacizumab treatment [120].

Ferroptosis resistance is similarly controlled by lactylation circuits. For example, HDAC1 K412 lactylation modulates RNA m6A via FTO/ALKBH5 to regulate ferroptosis sensitivity, establishing lactylation-mediated crosstalk between HDAC1 and m6A as a potential therapeutic target [121]. Furthermore, PRMT5 K240 lactylation represses ALKBH5, stabilizing SLC7A11 and reinforcing resistance to ferroptosis [122]. In CRC stem cells, lactylation at H4K12 by p300 upregulates GCLC, suppressing ferroptosis and sustaining chemoresistance [123].

Lactylation also amplifies key oncogenic transcriptional programs that support tumor growth. H3K18 lactylation boosts AURKB transcription, protecting PSAT1 mRNA from HNRNPM-mediated decay, thereby promoting serine/one-carbon metabolism and driving tumor growth [124]. Platelet-derived exosomal LINC00183 stabilizes ENO1, which further intensifies glycolysis and lactate production, amplifying the positive feedback loop that drives malignant progression [125].

Finally, lactylation also plays a role in metastatic spread. GPR37 activation promotes glycolysis and histone lactylation through the Hippo pathway, which increases CXCL1 and CXCL5 expression, driving liver metastasis [79]. In summary, lactylation drives CRC progression through a combination of metabolic reprogramming, enhanced DNA repair, redox regulation, and modulation of oncogenic transcription. These processes enable CRC cells to survive under therapeutic stress, resist chemotherapy, and metastasize, highlighting lactylation as a central player in CRC progression.

#### 4.1.2. Liver Cancer

Lactylation plays a crucial role in driving liver progression, including proliferation, metastasis, invasion, and resistance to chemotherapy, by modulating key molecular mechanisms within the TME. One of the central pathways involves lactate-induced histone lactylation, which promotes stress adaptation and metabolic rewiring. In the context of liver cancer, following sublethal microwave ablation, lactate-driven H3K18 lactylation upregulates NFS1, an enzyme involved in Fe–S biogenesis, to inhibit ferroptosis, thus enhancing metastatic potential and platinum resistance [126]. Other studies have shown that HCC cells exhibit lactylation at H3K9, K14, and K56, contributing to HCC progression [54,103,127].

Additionally, lactylation promotes EMT, a key process for tumor invasion and metastasis. K33 lactylation of TWIST1 enhances its nuclear activity, driving EMT and tumor progression [128]. This effect is further reinforced by lactylation’s ability to override tumor-suppressive redox regulation. For instance, SQLE-driven delactylation of CA3 reduces its function, restoring DUOX2 expression and enabling tumor cells to escape growth inhibition [129].

In terms of chemoresistance, lactylation modulates therapeutic responses by regulating key proteins involved in drug sensitivity. In liver cancer, histone lactylation increases the expression of USP34, a deubiquitinase, which stabilizes proteins that prevent cisplatin-induced cell death, promoting resistance to chemotherapy [130]. Furthermore, targeting lactylation has shown promise in overcoming resistance. For example, the triterpenoid demethylzeylasteral suppresses liver cancer stemness by inhibiting histone lactylation, which reduces H3K9la/H3K56la levels, linking lactate control to the epigenetic suppression of stem-like clones [103].

Beyond liver cancer, lactylation extends its influence on biliary tract cancers. In gallbladder cancer, lactylation activates the transcription of FBXO33, which polyubiquitinates p53, driving EMT and enhancing metastatic spread [131]. In intrahepatic cholangiocarcinoma (iCCA), lactylation of NCL at K477 by P300 enhances its interaction with the MAP kinase activating death domain (MADD) transcript, promoting ERK activation and tumor growth [24].

#### 4.1.3. Gastric Cancer

Lactylation plays a pivotal role in GC progression, particularly through its regulation of metabolic reprogramming, genomic stability, and resistance to therapeutic interventions. A mechanism involves the modulation of cuproptosis, a copper-dependent form of regulated cell death. Under copper stress, lactate-induced K229 lactylation of METTL16 enhances m6A methylation of FDX1 mRNA, triggering cuproptosis. However, SIRT2-mediated delactylation of METTL16 inhibits this process, with the combination of SIRT2 inhibition and a cuproptosis activator significantly suppressing GC cell proliferation [62].

Lactylation also influences oncogenic signaling pathways, including the H19-glycolysis-H3K18la feed-forward loop. Loss of SIRT1, a chromatin delactylase, releases this loop, leading to increased glycolysis and lactylation, accelerating gastric tumor progression [132]. Additionally, H3K18 lactylation enhances METTL14, which deposits m6A on ATF5, suppressing the ATF5/WDR74/β-catenin axis and reducing cancer stem-like traits [133]. Upstream, GLUT3 drives lactate production through LDHA, amplifying histone lactylation and further promoting GC progression. Depletion of GLUT3 dampens invasion and metastasis, indicating the importance of glycolytic flux in shaping the lactylome and tumor aggressiveness [134]. It is worth mentioning that PSMD14 promotes glycolysis and tumor progression in gastric adenocarcinoma (GAC) by deubiquitinating PFKFB2 at K355, enhancing its phosphorylation by SCYL2 at S466/S483, which increases fructose-2,6-bisphosphate levels and activates PFK1. This triggers a positive feedback loop involving H3K27 lactylation, which in turn upregulates the expression of PSMD14 and SOX9, contributing to poor prognosis in GAC [135]. It is also noteworthy that current omics data on histone H3 lactylation sites, such as H3K27 mentioned above, are relatively scarce. Currently, there is no experimental research linking H3K4 lactylation to tumor progression, which warrants further investigation.

#### 4.1.4. Esophageal Cancer

Lactylation plays a crucial role in driving the malignant progression of esophageal cancer (EC) by modulating metabolic reprogramming, stemness, and invasion through several molecular mechanisms. Under hypoxic conditions, lactate-induced histone lactylation stabilizes SHMT2, enhancing its interaction with MTHFD1L. This coupling of one-carbon metabolism with increased glycolysis promotes EC cell proliferation, invasion, and stemness. Additionally, hypoxia drives histone lactylation at H3K9, which in turn enhances the transcription of laminin subunit gamma 2 (LAMC2), contributing to tumor progression by promoting cell adhesion and invasion [53]. The lactylation of AXIN1 further modulates glycolysis by facilitating its ubiquitination, underscoring the importance of lactylation in metabolic control and its impact on the aggressiveness of EC [27].

### 4.2. Urinary and Reproductive System Cancers

#### 4.2.1. Bladder Cancer

Lactylation has emerged as a key regulator of bladder cancer (BCa) progression and therapeutic resistance through its effects on metabolic reprogramming, immune modulation, and genomic regulation. One of mechanisms involves the inhibition of HIPPO signaling by low CircXRN2 expression, which promotes enhanced glycolysis and subsequent H3K18 lactylation. This modification activates lipocalin 2 (LCN2) transcription, driving BCa progression. Notably, restoring CircXRN2 expression effectively suppresses BCa deterioration by reactivating the HIPPO pathway [136]. Lactylation also plays a crucial role in regulating resistance to chemotherapy in BCa. H3K18 lactylation drives the transcription of YBX1 and YY1, both of which are associated with cisplatin resistance in BCa, underscoring lactylation’s influence on drug resistance mechanisms [137]. Lactylation of YTHDC1 under high glucose conditions reduces BCa cell sensitivity to enfortumab vedotin (EV) by downregulating NECTIN4 expression, offering a potential strategy to overcome drug resistance through targeting lactylation [138].

Moreover, lactylation plays a role in reshaping the immune microenvironment. Mannose inhibits PKM2 lactylation, causing PKM2 to translocate to the nucleus, where it activates NF-κB and induces pyroptosis. This process enhances the efficacy of immune checkpoint inhibitors, suggesting a novel approach for boosting immune responses in BCa [139]. Additionally, hypoxia-induced PYCR1 enhances glycolysis and lactylation, driving BCa metastasis by activating the glutamine transporter SLC6A14. Silencing PYCR1, however, downregulates H3K18 lactylation and SLC6A14 expression, inhibiting tumor invasion [140]. Similarly, H3K18 lactylation promotes M2 macrophage polarization by activating PRKN-mediated mitophagy, contributing to an immune-suppressive TME that drives immune evasion and accelerates cancer progression [141].

#### 4.2.2. Prostate Cancer

Lactylation plays a central role in driving malignant progression, therapy resistance, and metabolic reprogramming in PC, with several key molecular mechanisms contributing to tumor growth, invasion, and metastasis. A prominent mechanism involves the H3K18 lactylation–NF-κB/p65–RPS6KC1–PRDX3 axis, which suppresses ferroptosis and promotes enzalutamide resistance. Targeting RPS6KC1 or inducing ferroptosis has been shown to restore drug sensitivity [142]. Therapeutic approaches targeting lactylation have shown promise in overcoming resistance. The combination of the PI3K inhibitor copanlisib and the Porcupine inhibitor LGK974 reduces H3K18 lactylation, restores macrophage phagocytic function, and inhibits the proliferation of PTEN/P53-deficient PC tumors [95]. Similarly, PI3K inhibition or PD-1 blockade has demonstrated the ability to suppress PC progression by targeting lactate-driven immune suppression and metabolic reprogramming [143]. In addition, chemical proteomics reveal that gambogic acid targets CNPY3 and recruits the delactylase SIRT1 to remove CNPY3 lysine lactylation, redirecting its localization to rupture lysosomes and induce pyroptosis—an essential mechanism to target in PC treatment [59].

In PC, defects in the NUMB/parkin RBR E3 ubiquitin ligase (PARKIN) pathway or overexpression of ZEB1 lead to metabolic reprogramming and the accumulation of lactate. This lactate promotes H3K18 lactylation, enhancing chromatin accessibility and cellular plasticity. This, in turn, upregulates the transcription of neuroendocrine-related genes such as MYCN and ASCL2, driving the transition from adenocarcinoma prostate cancer (ADPC) to neuroendocrine prostate cancer (NEPC) [144,145]. Furthermore, MCT1-mediated lactate influx stabilizes HIF-1α through lactylation, which transcriptionally upregulates KIAA1199. This enhances VEGFA secretion, VE-cadherin/p-EphA2 signaling, and hyaluronan remodeling, promoting angiogenesis and vasculogenic mimicry [146].

#### 4.2.3. Renal Cell Carcinoma

Lactylation plays a critical role in driving the progression of clear cell renal cell carcinoma (ccRCC) by modulating key cellular processes such as metabolic reprogramming, stress adaptation, and resistance to therapies. Under hypoxic conditions, p300-mediated K82 lactylation of the m6A reader YTHDC1 enhances its phase separation and nuclear condensate formation, protecting oncogenic transcripts (e.g., BCL2, E2F2) from decay, thereby promoting RCC progression [147]. This mechanism underscores lactylation’s impact on RNA stability and gene expression regulation in RCC. Additionally, lactylation accelerates glycolysis and mitochondrial function in RCC cells. FKBP10, through its PPIase domains, binds to LDHA and promotes its phosphorylation at Y10, amplifying the Warburg effect and increasing global histone lactylation. FKBP10’s activity is negatively regulated by HIFα, and its inhibition sensitizes RCC tumors to the HIF2α inhibitor PT2385, highlighting the importance of lactylation in metabolic shifts and therapy resistance [148]. In parallel, KAT8-mediated lactylation of MDH2 at K239 enhances its catalytic activity, coupling with the citrate carrier SLC25A1 to drive citrate efflux and NADPH production. This metabolic rewiring under high-lactate, low-glucose stress sustains tumor cell fitness by limiting ROS production, facilitating mitochondrial function, and promoting resistance to stress [149]. Finally, an oncogenic feedback loop connects inactive VHL-triggered histone lactylation to PDGFRβ transcription, further amplifying lactylation. Co-targeting lactylation and PDGFRβ signaling suppresses ccRCC growth and metastasis, suggesting that targeting this axis could be an effective therapeutic strategy [150].

#### 4.2.4. Cervical Cancer

Lactate secretion by CC cells has been shown to upregulate GPD2 expression, resulting in H3K18 lactylation in macrophages, which drives M2 macrophage polarization, which reshapes the TME, facilitating immune evasion and enhancing malignant transformation [151]. Furthermore, metabolic reprogramming in CC cells leads to lactate accumulation, which facilitates H3K14 lactylation. The reader of H3K14la, DPF2, binds to oncogene promoters, thereby driving transcription and ensuring cell survival, both of which are essential for tumorigenesis [152].

ICAT, which is over expressed in CC, plays a crucial role in enhancing the c-MYC-ENO1 axis, which increases glycolytic activity and lactate production. This, in turn, induces H3K18 lactylation in TAMs, contributing to an immunosuppressive M2 microenvironment that facilitates immune evasion and tumor progression [153]. Additionally, lactate-induced lactylation of DCBLD1 stabilizes its protein expression, activating the PPP, which promotes CC cell proliferation and metastasis by inhibiting G6PD autophagic degradation and enhancing PPP activity [69]. Together, these studies highlight how lactylation acts as a crucial molecular mechanism in the progression of cervical cancer, influencing not only the TME and immune response but also metabolic reprogramming and tumor cell survival.

#### 4.2.5. Ovarian Cancer

Recent studies have highlighted the role of lactylation in ovarian cancer (OC) chemoresistance, providing valuable insights into tumor metabolism and treatment resistance mechanisms. Lactylation of H3K18 on TRA2A facilitates the alternative splicing of STIL, suppressing ferroptosis and increasing resistance to cisplatin, a common chemotherapy drug [154]. Lactate accumulation triggers H4K12 lactylation, which activates super-enhancer-driven RAD23A expression, promoting niraparib resistance, thus exacerbating chemoresistance. A study investigating platinum resistance found that H3K9 lactylation activates RAD51 and BRCA2, enhancing homologous recombination repair and contributing to cisplatin resistance. This process can be reversed by inhibiting GCN5 [155].

Additionally, ACAT1-mediated acetylation of ME2 connects glutaminolysis to lactate production, enhancing chemoresistance by promoting lactylation of homologous recombination repair proteins [156]. In terms of immune evasion, LDHB regulates lactate production and induces H3K18 lactylation at the PD-L1 promoter, contributing to immune escape in OC [157]. A finding is that PFKP lactylation enhances glycolysis, promoting OC progression through the regulation of PTEN expression [158].

#### 4.2.6. Endometrial Cancer

Lactylation in the progression of endometrial cancer (EC), providing new insights into its contribution to tumorigenesis. One significant finding is that cold atmospheric plasma (CAP) induces ferroptosis in EC cells by activating the USP49/HDAC3 axis. This activation enhances lactylation-dependent P53 expression, ultimately inhibiting cell proliferation and migration [159]. Another pivotal mechanism revealed by recent research is that histone lactylation promotes EC progression by upregulating USP39 expression. This upregulation activates the PI3K/AKT/HIF-1α signaling pathway, which stabilizes PGK1, thus stimulating glycolysis and driving tumor cell proliferation, migration, and malignant transformation [160].

### 4.3. Endocrine System Cancers

#### 4.3.1. Breast Cancer

Lactylation, a post-translational modification of histones, plays a crucial role in breast cancer biology, influencing proliferation, migration, invasion, metastasis, and drug resistance. In cancer cells, enhanced glycolysis leads to lactate accumulation, which promotes histone lactylation, particularly at H4K12 and H3K18. This modification alters chromatin structure and affects oncogene expression. For example, H4K12 lactylation suppresses Schlafen 5 (SLFN5), promoting anti-apoptotic signaling and tumor growth in triple-negative breast cancer (TNBC) [161]. Lactylation also activates c-MYC, upregulating splicing factors like SRSF10, which drive alternative splicing of MDM4 and Bcl-x, promoting cell survival and chemotherapy resistance [162]. However, the precise mechanisms through which this epigenetic modification regulates gene activity remain to be fully elucidated. PWAS can systematically analyze the association between protein lactylation modifications and cancer phenotypes [163,164]. Nanoscopic imaging enables the visualization of the distribution of protein lactylation modifications at the single-cell or subcellular level [165]. Combining these approaches allows for a comprehensive understanding of the functional mechanisms of lactylation in breast cancer biology, from the macro (omics) level to the micro (subcellular) level.

Lactylation further influences DNA damage repair, contributing to therapy resistance. In TNBC, METTL3 lactylation by HDAC2 enhances DNA repair and cisplatin resistance [166]. Similarly, MRE11 lactylation via the HDAC5/HIF1α axis is essential for radioresistance, improving radiation response [167]. Additionally, lactylation of ZMIZ1 enhances its stability and transcriptional activity, driving tamoxifen resistance, tumor stemness, and cholesterol uptake [168]. These findings highlight lactylation’s central role in breast cancer resistance.

Targeting lactylation could be an effective therapeutic strategy. Inhibiting glycolytic enzymes or lactylation-specific pathways can reverse lactylation-induced oncogenic signaling, sensitizing tumors to chemotherapy and radiation [169]. Small molecules like MLN4924, which increase H3K18 lactylation and downregulate ITGB4, can reduce metastasis and migration in breast cancer cells [170]. High glucose conditions promote RCC2 lactylation, stabilizing MAD2L1 mRNA and driving tumorigenicity [171]. Additionally, lactate-induced lactylation enhances transcription factors regulating ferroptosis, offering new targets for overcoming chemotherapy resistance [172].

Lactylation also plays a critical role in metabolic reprogramming in breast cancer, particularly under anaerobic conditions. For instance, LDHC4 promotes the lactylation of ACAA2 at K214, enhancing its activity and increasing free fatty acid accumulation. This accelerates fatty acid metabolism, autophagy, and cell cycle progression, promoting TNBC progression [173]. in addition, histone lactylation regulates key transcription factors that facilitate cancer cell survival and proliferation. In breast cancer, H3K18la promotes PPARD expression, which activates the AKT pathway, supporting cell survival and proliferation during anaerobic glycolysis. This lactylation-mediated axis underscores the potential therapeutic value of targeting the H3K18la/PPARD/AKT pathway [174]. Moreover, lactylation affects metastasis. Hypoxia-induced lactylation of PRMT1 at K134/K145 enhances its methyltransferase activity, leading to asymmetric dimethylation of vimentin at R64. This promotes cytoskeletal reorganization and metastasis. Inhibition of PRMT1 with MS023 reduces metastasis in xenograft models, demonstrating lactylation’s role in metastasis regulation [175].

Research indicates that H3K18 lactylation is upregulated in breast cancer [162], while H4K12 lactylation is increased in TNBC [57]. Analysis of lactylation-associated genes in breast cancer suggests that these genes play a critical role in modulating tumor growth and the immune microenvironment [43].

#### 4.3.2. Pancreatic Cancer

Lactylation is closely tied to the metabolic reprogramming of cancer cells, particularly in the context of glycolysis. One of the key mechanisms involves the activation of α2δ1-driven calcium signaling, which enhances SIRT4 expression. SIRT4 deacetylates ENO1 at K358, increasing its affinity for 2-phosphoglycerate (2-PG), which boosts glycolysis and lactate production, which shift not only supports the proliferation of pancreatic cancer cells but also leads to broad histone lactylation at H3K9 and H3K18 [176].The interplay between lactate production and lactylation is central to the metabolic reprogramming that supports pancreatic cancer progression. Under hypoxic conditions, which are typical in PDAC, lactylation is dynamically regulated by enzymes like P300 and SIRT2. P300 facilitates histone lactylation at H3K18, while SIRT2 catalyzes the reverse reaction, contributing to a feedback loop that drives glycolysis and tumorigenesis. This metabolic-epigenetic feedback loop ensures the survival of cancer cells in hostile environments, promoting their ability to adapt and thrive despite metabolic stress [177].

High lactate levels, a hallmark of the TME, significantly contribute to the invasive properties of pancreatic cancer cells. Hyperglycemia, commonly seen in cancer, suppresses the activation of AMPK and promotes mitochondrial fission through DRP1-Ser 616, elevates lactate production, which in turn facilitates H3K18 lactylation and upregulates critical mitotic regulators like TTK and BUB1B. This lactate-driven activation accelerates pancreatic cancer progression by enhancing cell migration and invasion [178].

Lactylation also plays a crucial role in pancreatic cancer metastasis, particularly in perineural invasion. CAFs in the TME increase glycolysis and lactate production. This elevated lactate level induces H3K18 lactylation in tumor cells, which activates L1CAM and SLIT1-molecules that facilitate neural invasion, a key feature of pancreatic cancer metastasis. The lactate-induced epigenetic modification supports the metastatic spread of cancer cells through neural tissue, exacerbating the malignancy of the disease [179]. One of the most significant effects of lactylation is its contribution to chemotherapy resistance. In pancreatic cancer, lactate-induced H3K18 lactylation activates genes involved in cell survival and repair mechanisms, such as TTK, BUB1B, and MESP1, which are critical for maintaining cell-cycle progression and promoting chemoresistance. For instance, elevated levels of lactate enhance the lactylation of NMNAT1 at K128, sustaining the nuclear NAD(+) salvage pathway that supports cancer cell survival under glucose-deprived conditions, a common feature of tumors undergoing treatment [25].

### 4.4. Respiratory System Cancers

Respiratory system tumors mainly refer to lung cancer. Histone lactylation is a key regulator of lung cancer progression, influencing proliferation, migration, and invasion through metabolic reprogramming. In lung cancer, H4K8 and H4K16 are identified as primary histone lactylation sites. Lactylation of H4K8 regulates metabolism by promoting the transcription of HK1 and IDH3G [55], while serine/threonine kinase 11 (LKB1) inhibits lactylation at these sites [180]. In NSCLC, upregulation of NCAPD3 promotes tumor growth via the MEK/ERK/LDHA signaling axis, with histone lactylation enhancing NCAPD3 expression and amplifying glycolysis and malignant phenotypes [181]. Similarly, LDHA-mediated H3K18 lactylation drives glycolysis by regulating PTEN, facilitating cell proliferation and migration [182]. At the AIM2 locus, histone lactylation suppresses ferroptosis via ACSL4 and STAT5B, promoting tumor growth and invasion; inhibition of PKM2 or AIM2 reverses this effect [183]. Conversely, inhibition of lactate transport and lactylation through SLC4A7 limits lung adenocarcinoma progression by reducing lactate levels and lysine lactylation of oncogenes like HSP90AA1 [184]. The natural compound fargesin targets PKM2 to inhibit aerobic glycolysis and H3 histone lactylation, downregulating oncogenes and cell cycle regulators, thus providing a potential therapeutic approach for NSCLC [185].

In addition, NNMT (Nicotinamide N-methyltransferase) plays a critical role in acquired EGFR-TKI resistance in NSCLC. EGR1-mediated upregulation of NNMT triggers a feedback loop involving both epigenetic modifications and metabolic changes. Specifically, NNMT induces histone demethylation at H3K9me3 and H3K27me3, alongside lactate-induced H3K18 lactylation, establishing a positive feedback loop that reinforces EGFR-TKI resistance [186]. This feedback mechanism underscores the importance of lactylation and metabolic reprogramming in therapeutic resistance, revealing potential targets to overcome resistance in clinical settings.

Non-histone lactylation plays a critical role in lung cancer progression. In NSCLC, lactate induces lactylation of APOC2 at K70, which enhances its stability. This process leads to the release of FFAs, an accumulation of Treg in the TME, and resistance to anti-PD1 immunotherapy. Notably, using a custom anti-APOC2 K70-lac antibody can sensitize tumors to anti-PD1 immunotherapy [102]. Moreover, lactylation of SOX9 [187] and insulin like growth factor 1 receptor (IGF1R) [188] also contributes to the adverse progression of lung cancer.

### 4.5. Other Type Cancers

High levels of H3K18 lactylation is observed in glioblastoma, the NF-κB signaling pathway is a key factor driving H3K18 lactylation. H3K18 lactylation enhances the transcription of LINC01127 [189] and IL-10 [190], with LINC01127 promoting glioblastoma cell self-renewal and IL-10 suppressing T cell activity in the glioblastoma microenvironment. Additionally, in Treg cells co-cultured with glioblastoma cells, H3K18 lactylation enhances the transcription of genes such as C-C motif chemokine receptor 8 (CCR8) and ectonucleoside triphosphate diphosphohydrolase 1 (CD39), which diminishes the cytotoxic efficacy of CAR-T therapy against glioblastoma [191]. In endometrial cancer, H3K18 lactylation stimulates USP39 expression, thereby promoting cancer progression [160].

H3K18 lactylation also plays a significant role in melanoma progression. Enhanced H3K18 lactylation increases the expression of ALKBH3, a demethylase that removes m1A methylation from SP100A, a core component of tumor-suppressive promyelocytic leukemia bodies, this process facilitates malignant transformation in melanoma [21]. Conversely, H3K18 lactylation also upregulates YTH N6-methyladenosine RNA binding protein 2 (YTHDF2), which binds to m6A modification sites on period circadian regulator 1 (PER1) and TP53 RNA, promoting their degradation and exacerbating melanoma progression [20].

In AML, the highly expressed STAT5 mediates H4K5 lactylation. Under conditions of lactate accumulation, pyruvate dehydrogenase complex component X (E3BP) can translocate to the nucleus and enhance H4K5 lactylation, which increases the transcription levels of PD-L1 and contributes to the formation of an immunosuppressive microenvironment surrounding AML [22]. Mucin 20 inhibits the lactylation of IGF1R, thereby suppressing the activation of receptor tyrosine kinase. This inhibition increases the sensitivity of multiple myeloma cells to proteasome inhibitors (PIs) [192].

## 5. Blocking the Lactylation: A Potential Approach to Thwart Tumors Evolution

Although relatively systematic treatment methods exist for certain types of tumors, tumor cells have evolved strong adaptability through modifications to their surrounding microenvironment, significantly reducing the effectiveness of these therapies [193]. Lactate, as a primary covert agent, plays a crucial role in tumor resistance to treatment. Lactylation, which uses lactate as a substrate, directly participates in adaptive processes such as metabolic reprogramming and immune microenvironment remodeling in tumor cells. While numerous studies have focused on targeting lactate for tumor treatment [194], the feasibility of targeting lactylation for tumor therapy remains largely unknown. Excitingly, it has been reported that inhibiting the lactylase enzyme AARS1 with β-alanine effectively slowed tumor progression [18]. Additionally, a synthesized peptide that directly targets the lactylation of MRE11 at K673, when administered to mice at a non-toxic dose, this peptide significantly enhanced the sensitivity of tumor cells to chemotherapy drugs [30]. These pioneering studies offer a new perspective on cancer treatment: targeting protein lactylation as a therapeutic strategy.

Hypoxia and aerobic glycolysis are typical features of tumors, both hypoxic and aerobic regions often coexist within certain solid tumors [195]. Hypoxic tumor cells typically convert glucose into lactate through anaerobic glycolysis to obtain energy, with MCT4 overexpression facilitating the efflux of excess lactate [196]. This lactate can then be utilized by tumor cells in aerobic regions, which have high MCT1 expression [197]. Additionally, it can be taken up by other cell types within the TME, serving as a signaling molecule, an energy source, or mediating the lactylation of specific proteins, ultimately contributing to a tumor-promoting microenvironment [198]. Furthermore, the overexpression of GLUTs enhances the ability of tumor cells to efficiently uptake glucose from the surrounding environment under nutrient-deprived conditions [199,200]. Oncogenes such as HIF1α and c-MYC, along with signaling pathways aberrantly activated in tumors like PI3K-AKT, promote glycolysis by upregulating the transcription of enzymes including GLUTs, HK2, and PFK1 [201]. Excessive lactate can then act as a substrate for lactylation of glycolytic enzymes and upstream oncogenes, forming a feedback loop. Therefore, based on the above logic, this section primarily discusses methods of indirectly inhibiting protein lactylation, such as blocking lactate anabolism, promoting its catabolism, and inhibiting the lactate shuttle. It also explores representative potential strategies for directly targeting the lactylation of specific proteins for cancer therapy (Table 3). Many excellent reviews have already provided comprehensive summaries of lactate inhibitors, so we will not elaborate further here. It is important to note that while strategies for inhibiting lactate metabolism and protein lactylation share similarities, they are not entirely the same.

### 5.1. Blocking Lactate Anabolism/Promoting Lactate Catabolism

Inhibiting lactate anabolism could fundamentally alter the acidic microenvironment of tumors. Suppressing glucose transport (e.g., inhibiting GLUTs, particularly GLUT1), glycolytic enzymes, or LDHA can theoretically inhibit lactate anabolism. Natural compounds targeting GLUT1, such as silibinin [202] and genistein [203], as well as the glucose analog 2-DG (an HK2 inhibitor) [204], have been extensively studied in cancer therapy and are currently in Phase I/II clinical trials. LDHA inhibitors, including oxamate, GNE-140, and PSTMB [208,209], are also considered promising targets for cancer treatment. Moreover, it has been reported that the antiepileptic drug stridently reduces lactylation of NBS1 at K388 by inhibiting LDHA, thus overcoming chemoresistance in tumor cells [29]. Many other drugs targeting lactate synthesis are also under investigation [194].

Delivering natural enzymes, such as LOX and LDHB, to tumor tissues to promote lactate catabolism and inhibit lactylation is also a viable strategy [210,211,212]. LOX is an flavin mononucleotide (FMN)-dependent flavoprotein extensively studied in medical fields, including oncology, due to its close association with FMN and its ability to specifically catalyze the conversion of L-lactate to pyruvate and hydrogen peroxide using O_2_ as the electron acceptor [220,221,222], this makes LOX a promising candidate for future lactylation inhibition. In contrast, LDHB requires free NAD+ as an electron acceptor, which significantly limits its application compared to LOX [223]. Additionally, methods involving artificial nano enzymes and live bacteria to promote lactate catabolism are detailed in a review [224], so we will not elaborate further here.

It is important to note that due to the strong adaptability of tumor cells and the limitations of current lactate metabolism inhibitors-such as high systemic toxicity, low efficiency, high cost, and challenges in delivering them to effective sites-the effectiveness of using lactate anabolism inhibitors alone or solely delivering natural enzymes that promote lactate catabolism appears to be limited. Therefore, it is necessary to simultaneously inhibit lactate production and activate its elimination. The co-delivery of lactate anabolism inhibitors and lactate catabolism activators could more effectively mitigate the adverse effects of lactate on tumor progression [205,225,226].

### 5.2. Inhibiting Lactate Shuttle

In the organism, the transcellular transport of lactate mediated by MCTs is known as the lactate shuttle [227]. Tumor cells within different niches of solid tumors exhibit notable heterogeneity, and the lactate shuttle in the TME effectively promotes metabolic symbiosis among individual tumor cells under survival pressure—where tumor cells use lactate as a medium crosstalk with the microenvironment and share energy between individuals [228].

Lactate efflux from cells is mediated by MCT4, which is often abnormally upregulated in tumor cells due to hypoxia [229,230]. Additionally, some studies have reported that MCT4 regulates histone lactylation by facilitating lactate efflux [231,232,233]. Consequently, selective inhibitors of MCT4, including bindarit and its derivatives, as well as some nanomaterial-based inhibitors, may potentially inhibit lactylation [213,214,215]. Tumor cells expel excess lactate into the extracellular space via MCT4, which is then taken up and utilized by tumor cells with high MCT1 expression, typically those in oxygenated regions [197]. Additionally, other cell types within the TME also exhibit high MCT1 expression. The excessive uptake of lactate by these cells leads to increased histone lactylation, contributing to the development of an immunosuppressive TME [234]. High MCT1 expression in various cell types promotes protein lactylation [36,234,235,236]. Previously identified MCT1 inhibitors, such as novel substituted pteridine-derived inhibitors [216], BAY-8002 [217], coumarin carboxylic acids [218], and their analogs [219], are considered potential lactylation inhibitors. Notably, it has been demonstrated that the small-molecule MCT1 inhibitor 7ACC2 significantly reduced histone lactylation levels in NKT-like cells in vitro [234]. The expression of MCT1 and MCT4 in the TME is complex, posing a significant challenge for the precise targeting of these transporters.

### 5.3. Target Lactylation

Lactylation plays a significant role in tumor progression, with cancer cells driving malignant advancement through complex mechanisms that involve the regulation of protein lactylation [237]. Histone lactylation initiates the transcription of various oncogenes, and some oncoproteins are directly lactylated, which affects their stability and function. Protein lactylation has also been widely reported in diseases beyond cancer [238], highlighting the potential of targeting specific protein lactylation as a strategy for developing new therapeutic approaches for cancer and other conditions.

There are three main strategies for targeting lactylation: inhibiting lactyltransferases and delactylases, targeting lactyl groups and their donors, and focusing on specific protein core lactylation sites. First, directly inhibiting acyltransferases or activating deacylases could lead to cytotoxicity, as these enzymes transfer or remove a range of acyl groups, not specifically lactyl groups, making KATs and HDACs less suitable targets for lactylation. Recently discovered lactylases, AARS1 and AARS2, exhibit characteristics distinct from traditional KATs [18,23,44]. AARS1 was previously known as an ancient and conserved alanyl-tRNA synthetase. Recently, it has been discovered that AARS1 also functions as a lactate sensor and lactyltransferase. Its lactyltransferase activity can be effectively inhibited by exogenously supplied β-alanine, thereby suppressing lactylation [18,44]. Notably, exogenous β-alanine has also been reported to inhibit the malignant phenotype of breast cancer cells [239]. Therefore, targeting the delactylase activity of AARS1, in combination with chemotherapy and other therapies, presents a promising area for further research in cancer treatment. Targeting lactyl groups, particularly lactate, has been extensively discussed. Known lactyl donors include lactyl-CoA, lactyl-AMP, and lactyl-GSH. However, regulating intracellular levels of CoA, ATP, and GSH to target lactylation seems impractical, as these metabolites have numerous functions beyond serving as lactyl donors [240,241,242]. A cell-penetrating peptide that inhibits MRE11 K673 lactylation has been previously synthesized. Their research found that this peptide effectively suppresses homologous recombination repair and chemoresistance in tumor cells [30], suggesting that directly targeting the lactylation of specific proteins could be a promising approach for cancer therapy. Reports have shown that proteins closely related to tumors, such as P53 [18], YAP/TEAD [44], and HIF1-α [146], can undergo lactylation. Therefore, screening for or synthesizing small molecule inhibitors targeting the lactylation of key tumor-associated proteins, combined with other therapies, represents a promising direction for future research. Although lactylation at specific histone sites, particularly H3K18, plays a crucial role in tumor malignancy, the complex interactions between histones and DNA present significant challenges. Therefore, this topic will not be discussed further here. Although some have not yet been proven to inhibit lactylation, they are considered likely to have such effects.

## 6. Conclusions and Future Directions

Lactylation is a recently identified post-translational modification that uses substrates like lactate and coenzyme A under the catalysis of acyltransferases; lactyl groups are attached to lysine residues on substrate proteins. An increasing number of reports indicate that lactylation acts as a mediator between epigenetic modifications and gene transcription regulation, playing a crucial role in modulating protein stability and function. The discovery of lactylation provides a novel perspective on the role of lactate in tumor metabolic reprogramming and microenvironment remodeling and presents a promising target for inhibiting tumorigenesis, metastasis, and chemotherapy resistance.

The question of whether KATs and lactyl-CoA are the primary lactyltransferases and lactyl donors, respectively, or whether AARS and lactyl-AMP play a more significant role, or if these two forms of lactylation function collaboratively within this complex microenvironment, still needs to be clarified. Furthermore, current research on histone lactylation primarily focuses on specific sites, including H3K9, H3K18, H4K8, and H4K12. However, few studies have investigated other histone lactylation sites, which require further investigation. In the field of lactylation-regulated tumor metabolic reprogramming, it is well-established that lactylation closely interacts with glycolysis. However, proteomic analyses have reported significant enrichment of lactylation in processes such as amino acid metabolism, nucleotide metabolism, lipid metabolism, and the tricarboxylic acid cycle [15,233,243]. Nevertheless, relatively few studies have explored lactylation in these biological processes. Finally, targeting AARS to regulate global lactylation represents a promising research direction. Developing targeted therapies against specific proteins for cancer and other diseases is highly warranted. This paper provides the most comprehensive review to date of lactylation, covering its biological principles, role in regulating tumor metabolic reprogramming and microenvironment remodeling, promotion of tumor progression, and potential strategies for targeting lactylation in cancer therapy. The aim of this review is to offer researchers a theoretical foundation and direction for future studies in this field.

## Figures and Tables

**Table 1:** Part of representative downstream genes regulated by histone lactylation, their effects, and the types of diseases they influence.

Site	Upregulated Genes	Effect	Refs
H3K18	*YTHDF2*, *ALKBH3*, *circATXN7*	Promote tumor proliferation, migration, invasion, metastasis, chemotherapy resistance	[20,21,51]
H3K9	*LUC7L2*, *LAMC2*, *ESM1*	Regulates tumor development	[52,53,54]
H4K8	*HK1*, *IDH3G*, *LINC00152*	Regulates cellular metabolism, invasion and migration	[55,56]
H4K12	*CCNB1*, *ABCs*	Promotes proliferation and chemotherapy resistance	[57,58]

**Table 2:** Representative lactylated proteins in tumors, including their lactylation sites and the functional consequences of lactylation.

Name	Lactylation Site	Effects	Refs
MOESIN	K72	Strengthen their own typical function	[65]
MRE11	K673		[30]
eEF1A2	K408		[66]
NCL	K477		[24]
YAP	K90		[44]
TEAD	K108		[44]
NSUN2	K356		[67]
NBS1	K388		[29]
METTL16	K229		[62]
VPS34	K356/K781		[40]
CCNE2	K348		[64]
CENPA	K124		[68]
DCBLD1	K172	Maintain its own stability	[69]
NUSAP1	K34	Inhibit self-degradation	[26]
AXIN1	K147	Promote ubiquitination	[27]
NMNAT1	K128	Enhance enzyme activity	[25]

**Table 3:** Some representative examples of strategies to block lactylation in tumors.

Strategy	Target	Inhibitor	Refs
Block lactate anabolism	GLUT1	Silibinin (C_25_H_22_O_10_)	[202]
		Genistein (C_15_H_10_O_5_)	[203]
	HK2	2-DG (C_6_H_12_O_5_)	[204]
	PFK	3PO (C_12_H_8_N_2_O)	[205]
		PFK15 (C_17_H_12_N_2_O)	[206]
	PKM2	Benserazide (C_10_H_14_N_2_O_4_)	[207]
	LDHA	Oxamate (C_2_H_2_NO_3_^−^)	[208,209]
		GNE-140 (C_25_H_23_ClN_2_O_3_S_2_)	
		PSTMB (C_13_H_9_F_3_Se)	
Promote lactate catabolism	LOX	Natural lactate oxidase (LOX)	[210,211]
	LDH	Selenide nanosheets	[212]
Inhibit lactate Shuttle	MCT4	Bindarit (C_19_H_20_N_2_O_3_) and its derivatives	[213]
		Nanomaterial-based inhibitors	[214,215]
	MCT1	Novel substituted pteridine-derived inhibitors	[216]
		BAY-8002 (C_20_H_14_ClNO_5_S)	[217]
		Coumarin carboxylic acids (C_10_H_6_O_4_)	[218,219]
Target lactylation	AARS1	β-alanine	[18]
	MRE11	Cell-penetrating peptide	[30]

## Data Availability

No new data were created or analyzed in this study.
